# Hyaluronan promotes TRPV4-induced chondrogenesis in ATDC5 cells

**DOI:** 10.1371/journal.pone.0219492

**Published:** 2019-08-08

**Authors:** Yoshikazu Ogawa, Nobunori Takahashi, Toki Takemoto, Tsuyoshi Nishiume, Mochihito Suzuki, Naoki Ishiguro, Toshihisa Kojima

**Affiliations:** Department of Orthopaedic Surgery, Nagoya University Graduate School of Medicine, Tsurumai, Showa-ku, Nagoya, Japan; University of Rochester, UNITED STATES

## Abstract

Hyaluronan (HA) is an extracellular matrix glycosaminoglycan essential for the homeostasis of cartilage-related tissues. Intracellular adhesion molecule-1 (ICAM-1) and CD44 have been identified as receptors for HA. Recently, transient receptor potential vanilloid 4 (TRPV4) has emerged as a potential research target in several areas of physiology. TRPV4 is a Ca^2+^-permeable, non-selective cation channel that appears to have mechanosensory or osmosensory roles in several musculoskeletal tissues. HA and TRPV4 play key roles in chondrogenesis; however, it has remained unclear whether they have interactive effects on chondrogenesis and, if so, how do they interact with each other? This study investigated the relationship between HA, its receptors ICAM-1 and CD44, and TRPV4 in the chondrogenic pathway using the ATDC5 cell line. It was found that the presence of HA is required for TRPV4-induced chondrogenesis. Loss of HA suppressed TRPV4-induced expression of the chondrogenic markers, SOX9 and Aggrecan. Moreover, HA affects TRPV4-induced chondrogenic development via each of ICAM-1 and CD44 partially. In conclusion, for the first time, the existence of an interaction between HA, its receptor ICAM-1 and CD44, and TRPV4-activity in chondrogenesis in the ATDC5 cell line was reported. TRPV4 is known to function as a mechanosensory channel in several musculoskeletal tissues. Therefore, findings of this study may suggest the existence of a molecular mechanism that underlies the interactive effects of HA and mechanical loading on joint chondrogenesis.

## Introduction

Hyaluronan (HA) is an extracellular matrix glycosaminoglycan composed of repeating disaccharides of glucuronic acid and *N*-acetylglucosamine [1, 2]. HA plays an essential role in the homeostasis of cartilage-related tissues [3–5], suggesting that it is required for chondrogenesis. It also protects human osteoarthritic chondrocytes by reducing oxidative stress, apoptosis, and mediators of inflammation and catabolism in those tissues [6]. Moreover, intra-articular administration of HA increases the volume of hyaline cartilage [7]. Intracellular adhesion molecule-1 (ICAM-1) and CD44 have been identified as the major receptors for HA [8–10]. CD44 is a major cell surface receptor for HA, and its interactions with HA play important roles in various biological events, including cell proliferation and metastasis [11]. Furthermore, recent studies have indicated the direct involvement of ICAM-1 in the biological action of HA [12]. Articular chondrocytes constitutively express ICAM-1 and CD44 [13]. HA can enter cartilage and bind to ICAM-1 on chondrocytes [14]. The association between HA and ICAM-1 can elicit IL-1, FN-f, and lipopolysaccharide effects [8, 15, 16].

Recently, transient receptor potential vanilloid 4 (TRPV4) has emerged as an important target in diverse research fields [17–19]. TRPV4 is a Ca^2+^-permeable, non-selective cation channel that appears to have mechanosensory or osmosensory roles in various musculoskeletal tissues [20]. TRPV4 provides an essential physiological link between mechanical loading and chondrocyte functioning by regulating extracellular matrix biosynthesis in cartilage [21]. The presence of HA and TRPV4 activity affect chondrocyte functioning. However, no data are available as to whether the effects induced by HA interact with those induced by TRPV4 activation during chondrogenesis. Therefore, the present study aimed to investigate whether pericellular HA was involved in chondrogenesis induced by TRPV4 activation.

## Materials and methods

### Cell culture

The ATDC5 cell line is a continuous, long-term cultured line derived from mouse teratocarcinoma cells and is commonly used as a model for *in vitro* chondrocyte research. ATDC5 cells, purchased from Riken Cell Bank (Ibaraki, Japan), were plated at a density of 1 × 10^5^ cells/well in a 6-well plate. These cells were cultured in 5% CO_2_ and 21% O_2_ at 37°C for 48 h in Dulbecco’s modified Eagle medium/Nutrient F-12 Ham (Sigma, MO, USA), containing 5% fetal bovine serum and 1% antibiotic-antimycotics. Then, at approximately 80% confluence, the cells were supplemented with 1% insulin-transferrin-sodium selenite (ITS) (Sigma, MO, USA) to support chondrogenic differentiation at 48 h prior to initiation of the experimental conditions.

### TRPV4 activation

To determine the effect of TRPV4 activation on the expression of chondrogenic markers, ATDC5 cells were stimulated with 10–10000 nM of the selective TRPV4 agonist, GSK1016790A (GSK) (Sigma, MO, USA), or with a vehicle control, dimethyl sulfoxide (DMSO), for 6–72 h in serum-free medium followed by incubation with 1% ITS for 48 h. The mRNA expression levels of SOX9, Aggrecan, type 2 collagen, and TRPV4 were quantitatively measured by real-time PCR.

### Influence of extracellular HA degradation on TRPV4-induced chondrogenesis

To determine the effect of removal of the extracellular matrix HA on the expression of chondrogenic markers following 48 h incubation with 1% ITS, ATDC5 cells were pretreated for 12 h with 0–100 nM of hyaluronidase from *Streptomyces hyalurolyticus* (HAase) (Sigma, MO, USA) to generate “matrix-depleted” cells. These cells were then rinsed twice in PBS and stimulated with 100 nM of GSK for 6 and 24 h to assess the mRNA expression levels of SOX9 and Aggrecan, respectively.

In the HA-rescue protocol, to confirm the impact of extracellular HA on chondrogenesis, HAase-treated medium as described above was subsequently replaced by HAase-free medium with 2 mg/ml of high molecular-weight HA (Artz, molecular weight: 600–1200 kDa; Seikagaku Corporation, Japan) for 1 h prior to TRPV4 activation with 100 nM of GSK.

### Neutralization against HA receptors CD44 and ICAM-1

In order to elucidate the role of the HA receptors CD44 and ICAM-1 in the chondrogenic pathway, ATDC5 cells were pretreated with a monoclonal neutralizing antibody against CD44 (1:100 or 1:1000; ab119335, Abcam, UK), ICAM-1 (1:100 or 1:1000; ab171123, Abcam, UK), or a purified IgG isotype control antibody (BioLegend, CA, USA) for 1 h. This was followed by incubation with or without 100 nM of GSK for 6 and 24 h to obtain SOX9 and Aggrecan mRNA, respectively. Lysate collection for western blotting analysis of SOX9 and Aggrecan was performed at 12 h after the respective mRNA collections.

### Real-time reverse transcription-polymerase chain reaction (real-time PCR)

Total RNA was isolated from the ATDC5 cultures using the RNeasy Mini Kit (Qiagen, Germany) according to the manufacturer’s instructions. RNA was reverse-transcribed using High-Capacity cDNA Reverse Transcription Kits (Applied Biosystems, US) with RNase inhibitor. For real-time PCR, the PCR products were detected using LightCycler 480 SYBR Green I Master (Roche, Switzerland). The primer mouse-specific sequences were GAPDH, forward (5′- GCCAGCCTCGTCCCGTAG -3′) and reverse (5′-TGAGGTCAATGAAGGGGTCGTT-3′); SOX9, forward (5′- CGACTCCCCACATTCCTCCTC -3′) and reverse (5′- GGACCCTGAGATTGCCCAGAG -3′); and Aggrecan, forward (5′- CAGCAGAAACAACCATGTCCCT -3′) and reverse (5′- CCTCACATTGCTCCTGGTCTG -3′). Thermal cycling and fluorescence detection were performed using a LightCycler 480 Smart system (Roche, Switzerland). Changes in mRNA expression levels of target genes were calculated as relative ratios, using GAPDH expression levels as internal controls.

### Western blotting analysis

Cells were harvested in RIPA lysis buffer (sc-24948, Santa Cruz Biotechnology, CA, USA). Total protein lysate was mixed with LDS Sample Buffer and Sample-Reducing Agent (Thermo Fisher Scientific, CA, USA) and denatured at 70°C for 10 min. SDS-PAGE (4%–12%) gels (Thermo Fisher Scientific, CA, USA) were used to separate 20 μg of whole-cell lysate under denaturing conditions; then, a wet electroblotting apparatus (Bio-Rad, CA, USA) was used to transfer the lysate to a polyvinylidene-difluoride membrane (Merck Millipore, Germany). The membranes were blocked for 1 h at room temperature in 5% non-fat dry milk (Cell Signaling Technology, MA, USA) in TBS (0.125 M Tris, 0.75 M NaCl; pH = 7.4) and probed overnight at 4°C with specific antibodies against SOX9 (1:200; ab3697, Abcam, UK), Aggrecan (1:500; AB1031, Merck Millipore, Germany), or β-actin (1:1000; #4970, Cell Signaling Technology, MA, USA) diluted in Immunoreaction Enhancer Solution (Toyobo, Japan). After three washes in TBS with 0.1% Tween 20, the membranes were incubated at room temperature for 1 h with secondary antibody goat anti-rabbit IgG (1:2000; #7074, Cell Signaling Technology, MA, USA), conjugated with horseradish peroxidase. Finally, membranes were washed three times for 5 min each in TBS with 0.1% Tween 20, then developed using Super Signal West Pico Chemiluminescent Substrate (Thermo Scientific, MA, USA), and visualized using an Ez-Capture-2 CCD camera (Atto, Japan). Band intensities were captured with a digital image scanner and quantified using densitometry software (CS Analyzer 3.0; ATTO, Tokyo, Japan).

### Alcian blue histochemical staining and quantification

Cells were fixed with 100% methanol at -20ºC for two minutes, followed by incubation with 0.1% Alcian Blue (Sigma-Aldrich, St. Louis, USA) for two hours at 25ºC [22]. Samples were washed three times with distilled water and observed with a microscope. Since Alcian Blue is used to stain acidic polysaccharides such as glycosaminoglycan, which is representative of cartilage matrix, we measured the amount of extracted blue dye as an index of chondrogenic differentiation. Stained cells were directly extracted with 1 ml of 6M guanidine-HCl for eight hours at 25ºC. Total optical density of the extracted dye in 6M guanidine-HCl at 620 nm was measured with SUNRISE (TECAN, Männedorf, Switzerland).

### Statistical analysis

Data were represented as mean ± SEM (standard error of the mean). Each experiment was performed six times; the reported relative expression values therefore represent the mean outcomes of six trials. Statistical analyses were performed using one-way or two-way analysis of variance (ANOVA) followed by post-hoc Bonferroni tests to compare differences between the various treatment groups as indicated in the figure legend. P < 0.05 was considered statistically significant.

## Results

### TRPV4 activation by the specific small molecule activator, GSK, stimulates in vitro expression of chondrogenic markers

**Fig 1:**
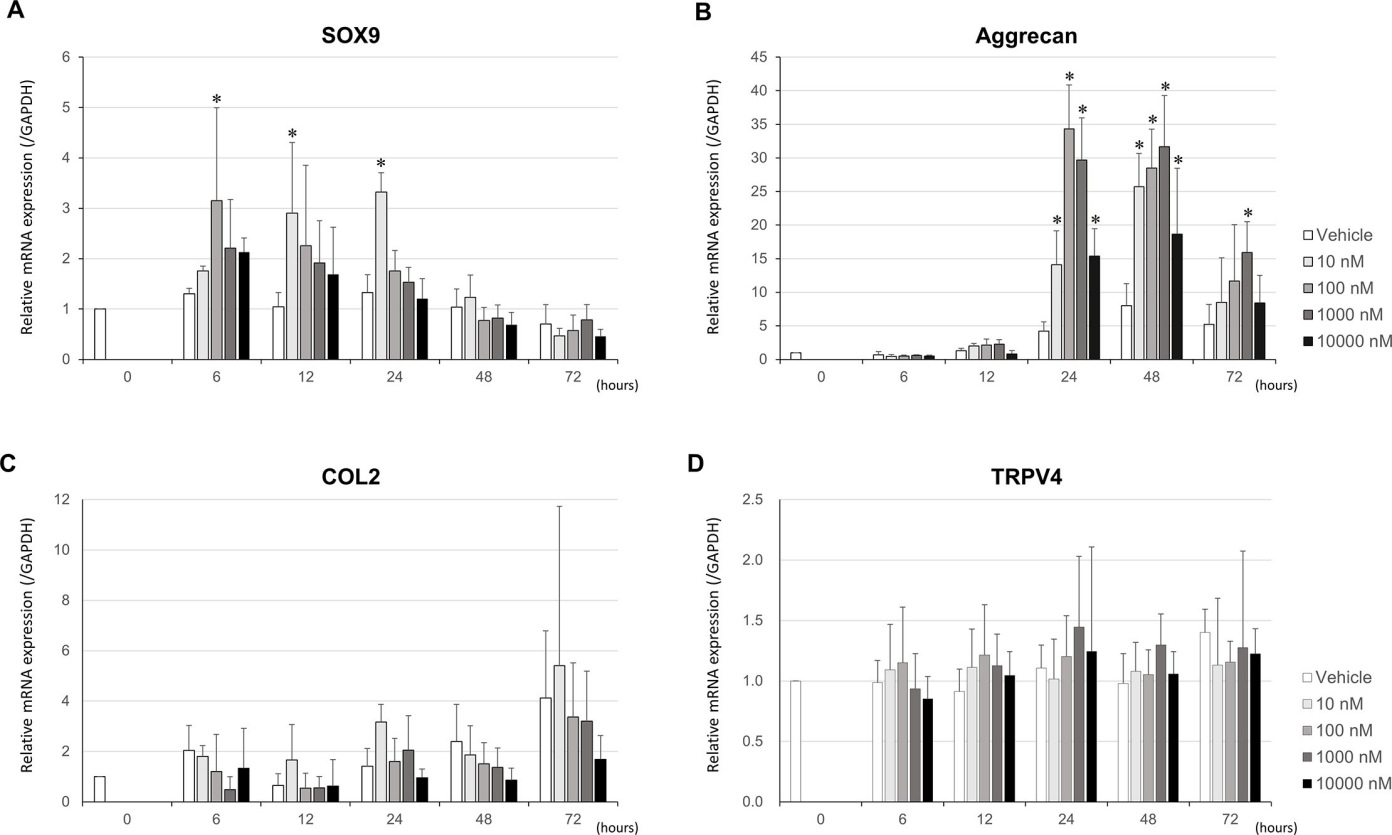
TRPV4-dependent mRNA expression levels in ATDC5 cells following treatment with the TRPV4-agonist, GSK1016790A. The TRPV4-specific, small molecule activator GSK accelerates the *in vitro* expression of chondrogenic markers. (A and B) GSK enhances mRNA expressions of SOX9 and Aggrecan in a time- and dose-dependent manner. (C) Type 2 collagen (COL2) expression was slightly increased by GSK stimulation but difference was not statistically significant. (D) TRPV4 expression was not affected by GSK stimulation. The mRNA expression of target genes of interest is normalized to that of the house-keeping gene, GAPDH. *greater than vehicle control at each time point (two-way ANOVA, P < 0.05; N = 6). Values are expressed as mean ± SD. Statistical significance is estimated by two-way ANOVA followed by post-hoc Bonferroni test.

GSK facilitated the expression of chondrogenic markers, as measured by the mRNA levels of SOX9 and Aggrecan, in ATDC5 cells by TRPV4 activation in a dose- and a time-dependent manner (Fig 1A and 1B). The expression levels of type 2 collagen (COL2) was slightly increased with GSK stimulation but the difference was not statistically significant (Fig 1C). We decided therefore to study the expression levels of SOX9 and aggrecan as the representative genes evaluating chondrogenesis progression. The expression levels of TRPV4 were quite consistent and were not affected by the GSK stimulation (Fig 1D). In this experiment, 100 nM of GSK most prominently induced mRNA expression of SOX9 at 6 hours and Aggrecan at 24 hours, respectively; therefore, these different time courses were used for each mRNA expression measurement in this study. The mRNA extractions were performed at 6 hours for SOX9 and at 24 hours for Aggrecan after the GSK stimulation started.

Cell viability was determined by MTS assay, using Celltiter 96 Aqueous One Solution Cell Proliferation Assay (Promega). ATDC5 cells were seeded in 96 well plate so that 5,000 cells were included per well and incubated in DMEN/F12 containing 5% FBS in 5% CO_2_ atmosphere for 24 hours. Cells were exposed to 100 μl of serum free medium containing different concentration of GSK101 and 0.1% DMSO. After 24, 48, and 72 hours, MTS assay was performed in according to Promega’s product protocol. Absorbance at 490 nm wave length was recorded on a microplate reader. Incubation with 10000 nM of GSK for 48 and 72 hours significantly decreased the cell viability. However, 100 nM of GSK for 6 or 24 hours did not affect the cell viability (S1 Fig).

### Effect of another TRPV4 chemical activator on SOX9 expression

**Fig 2:**
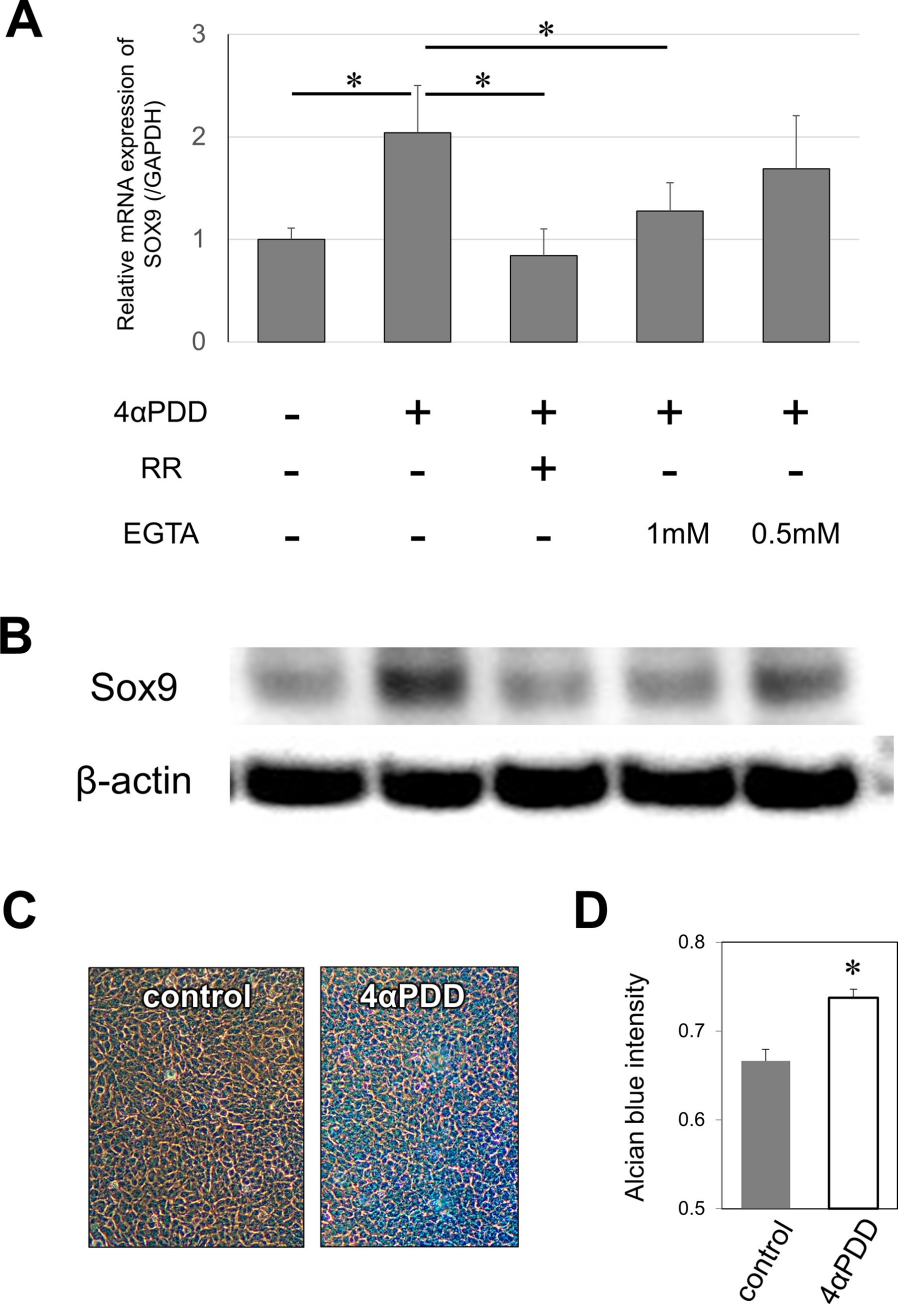
Effect of chemical TRPV4 activator, 4αPDD, on chondrogenesis in ATDC5 cells. Prior to treatment with 4αPDD, cells were treated with RR or EGTA. (A) Sox9 mRNA (n = 3) and (B) protein expression were assessed on day 1. (C) Cells were stained with Alcian Blue on day 7 and (D) the concentration of extracted dye was measured at 620 nm. Total optical density of extracted dye of ATDC5 cells was used as an index representing the amount of Alcian Blue. *4αPDD*: 4α-phorbol 12,13-didecanoate, *RR*: ruthenium red. Values are mean ± S.D. *p<0.05, vs cells with 4αPDD supplementation, Student’s t-test for two-group comparisons and Bonferroni/Dunn analysis for multiple-group comparisons.

Next, we determined to confirm whether chemical activation of TRPV4 via the specific synthetic activator 4α-phorbol 12,13-didecanoate (4αPDD) (Calbiochem, La Jolla, USA) can induce chondrogenesis [23]. To inhibit stimulation of the TRPV4 pathway, we used ruthenium red (RR) (Calbiochem), a TRPV family antagonist [23], and EGTA (SIGMA), a calcium chelator. Confluent ATDC5 cells on 6-well plastic culture plates were incubated with ITS in the presence or absence of 360 nM 4αPDD for 24 hours. The mRNA expression levels of Sox9 were significantly higher in cells treated with 4αPDD (p = 0.002) (Fig 2A). Another set of cells were pre-treated with RR or various concentrations of EGTA for 0.5 hours before treatment with 4αPDD. The increasing of Sox9 mRNA expression by 4αPDD was significantly reduced by pre-treatment with 10 μM RR (p = 0.002) and 1 mM (p = 0.006) EGTA, but not significantly by 0.5 mM EGTA (p = 0.24) (Fig 2A). Similar effects on Sox9 expression were also confirmed by western blotting analysis (Fig 2B). Alcian Blue staining was performed on cells treated for seven days with or without 4αPDD (Fig 2C). Cells treated with 4αPDD exhibited significantly higher GAG accumulation compared to cells treated without the activator (p = 0.002) (Fig 2D).

### Extracellular HA degradation attenuates TRPV4-induced expression of chondrogenic markers

**Fig 3:**
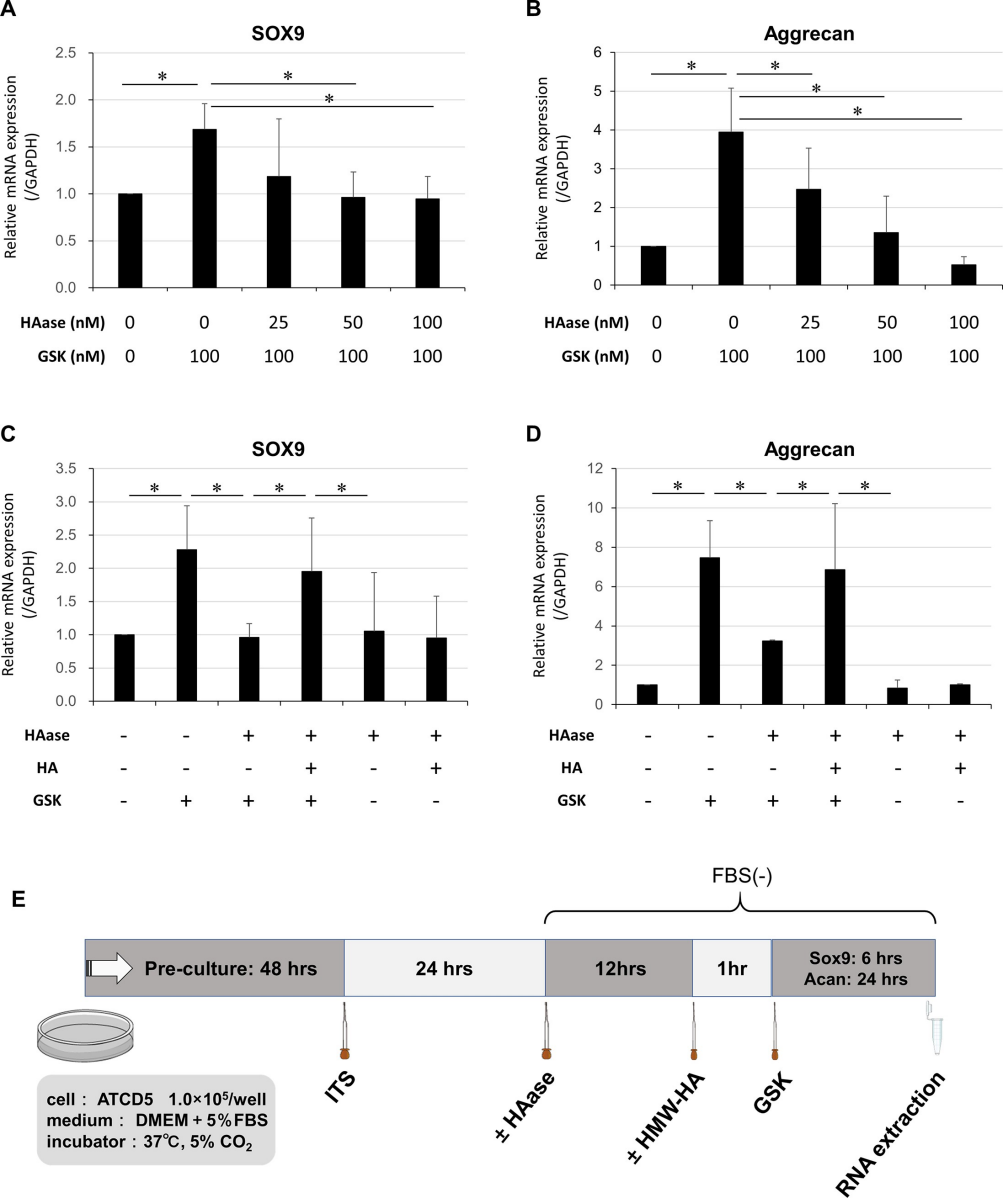
Effect of HA degradation on TRPV4-induced expression of chondrogenic markers as measured by quantitative RT-PCR. (A and B) HA degradation by HAase dose-dependently suppressed TRPV4-induced expression of chondrogenic markers. (C and D) Restored expression of chondrogenic markers when 2 mg/ml of HA was added after prior HA degradation by 50 U/ml of HAase. The mRNA expression levels of SOX9 and Aggrecan are shown as values relative to the levels of GAPDH. (E) Time diagram of treatments. *greater than controls (one-way ANOVA, P < 0.05; N = 6). Values are expressed as mean ± SD. Statistical significance is estimated by one-way ANOVA followed by post-hoc Bonferroni test. HA: hyaluronan, HAase: hyaluronidase, FBS: fetal bovine serum.

**Fig 4:**
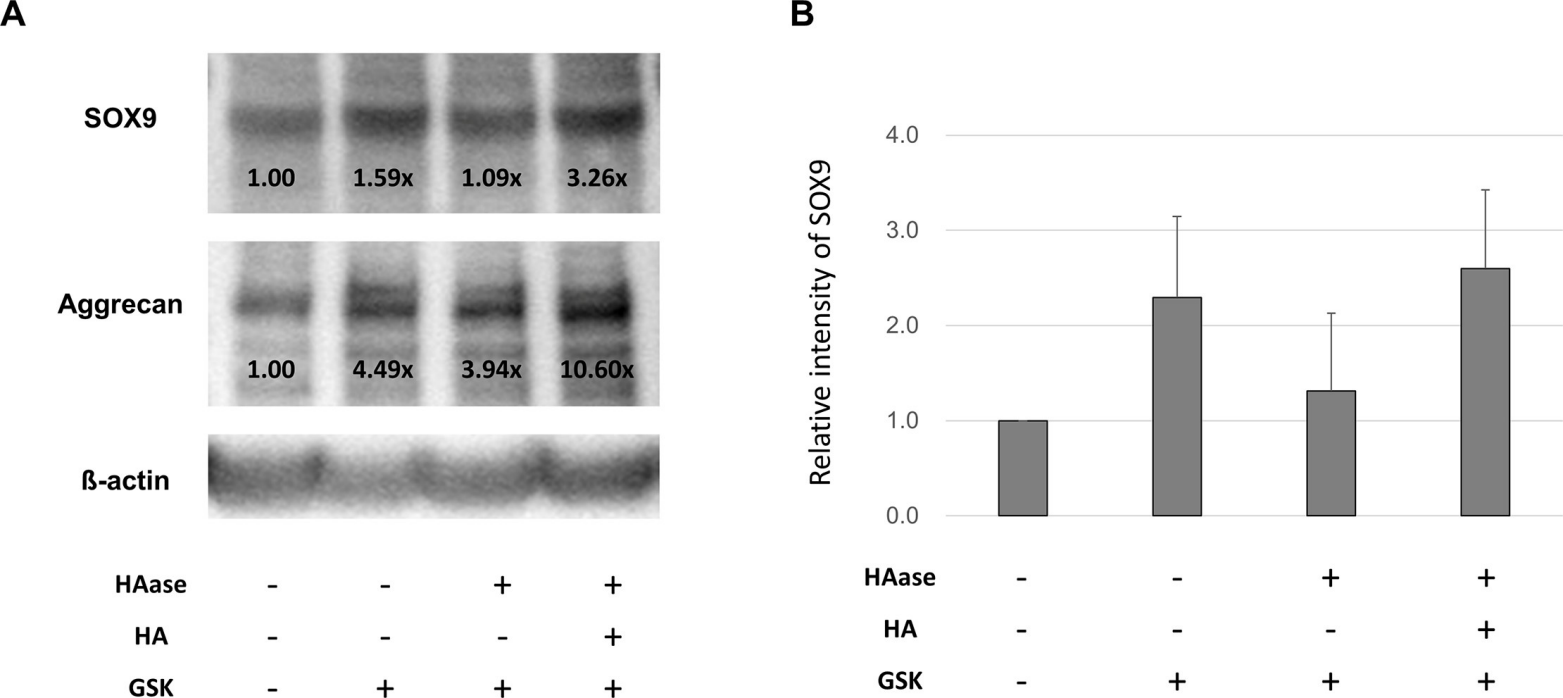
Effect of HA degradation on TRPV4-induced protein expression of SOX9 and *Aggrecan* examined by western blotting analysis. (A) Addition of 2 mg/ml of HA restored the expression levels of chondrogenic markers, which had been previously suppressed by degradation of HA with 50 U/ml of HAase. The GSK concentration used above was 100 nM. Values determined by densitometry show the fold-change in SOX9 and Aggrecan band intensity of each samples. (B) Densitometry of SOX9. Values are mean ± SD. HA: hyaluronan, HAase: hyaluronidase.

The extent of chondrogenesis, as determined by the mRNA expression levels of SOX9 and Aggrecan, was inversely correlated with the concentration of HAase in ATDC5 cells (Fig 3A and 3B). Without the GSK stimulation, HAase treatment alone did not affect the expression of chondrogenic markers, suggesting that HAase treatment itself has no effect on chondrogenesis (second bars from right in Fig 3C and 3D). Furthermore, supplementation with high molecular weight HA (HMW-HA) after HAase treatment was found to substantially restore mRNA levels of SOX9 and aggrecan (Fig 3C and 3D). HAase treatment and subsequent reintroduction of HA without TRPV4 stimulation did not restore the mRNA expression levels of these chondrogenic markers. Thus, the above findings provide evidence that pericellular HA is required for chondrogenesis elicited by TRPV4 activation. The protein levels of SOX9 and aggrecan were determined by western blotting analysis (Fig 4A).

### HA regulates TRPV4-induced chondrogenesis via ICAM-1 rather than CD44

**Fig 5:**
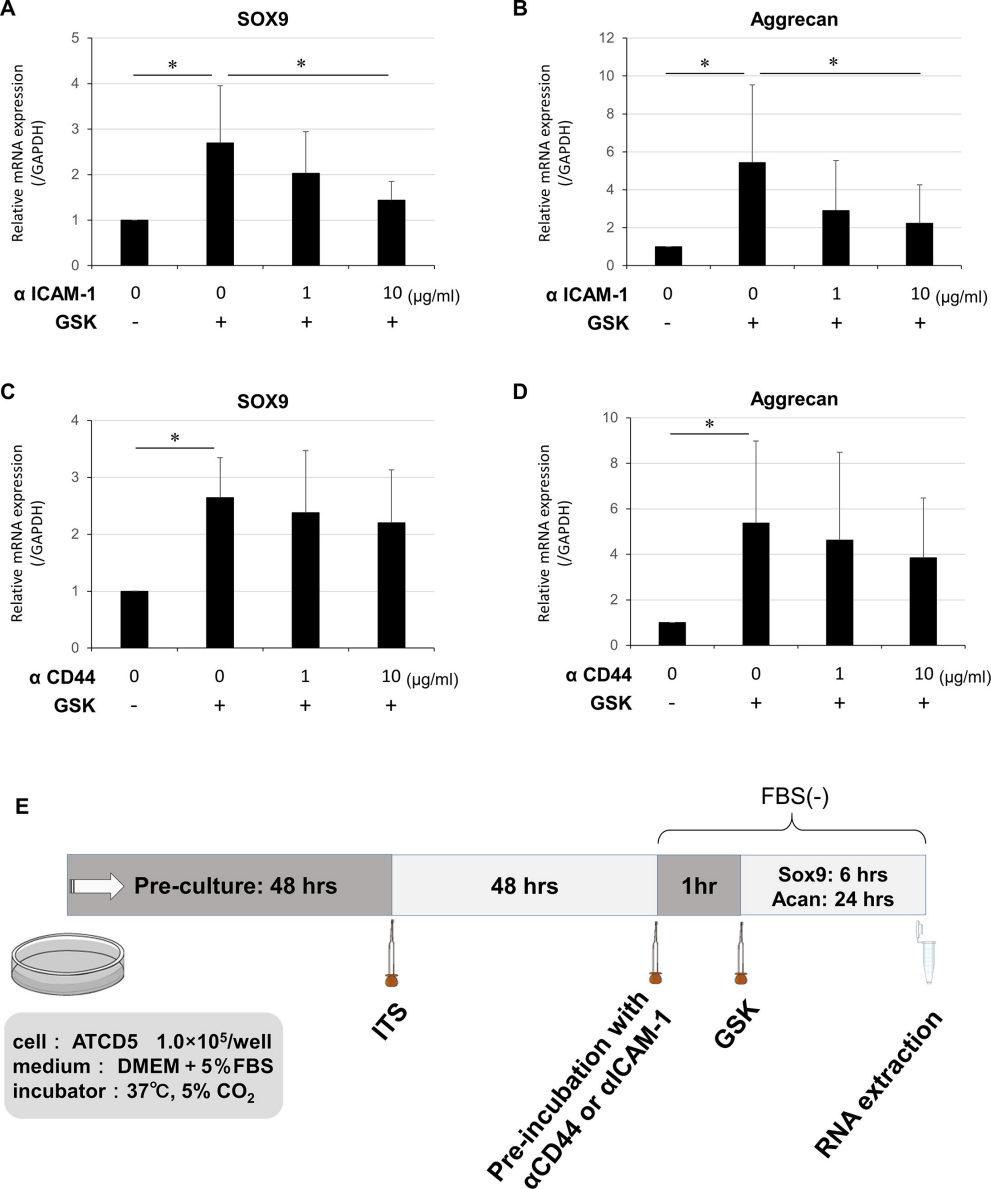
Effect of neutralization of HA receptors on TRPV4-induced expression of chondrogenic markers. (A and B) TRPV4-induced mRNA expression levels were dose-dependently suppressed by monoclonal neutralizing antibodies against ICAM-1. (C and D) The effect of antibodies against CD44 was similar but milder than that of ICAM-1. The mRNA expression levels of SOX9 and Aggrecan are shown as values relative to the levels of GAPDH. (E) Time diagram of treatments. *greater than control (one-way ANOVA, P < 0.05; N = 6). Values are expressed as mean ± SD. Statistical significance is estimated by one-way ANOVA followed by post-hoc Bonferroni test. αICAM-1: anti-ICAM-1 neutralizing monoclonal antibody, αCD44: anti-CD44 neutralizing monoclonal antibody, FBS: fetal bovine serum.

**Fig 6:**
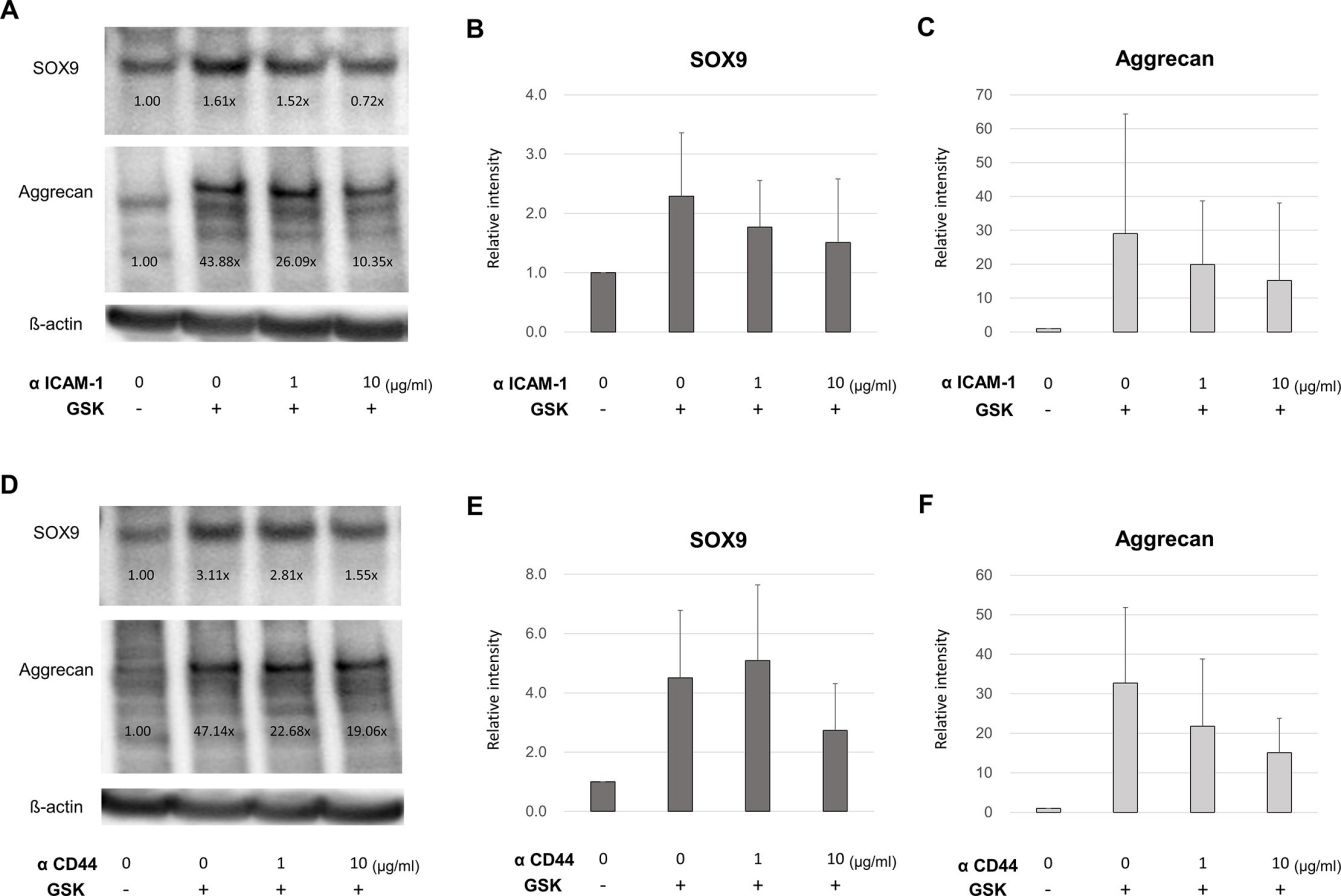
Effect of neutralization of HA receptors on TRPV4-induced protein expression of SOX9 and *Aggrecan* examined by western blotting analysis. (A and D) anti-ICAM-1 and anti-CD44 neutralizing antibodies decreased the TRPV4-induced protein expressions of SOX9 and Aggrecan in a dose-dependent manner. The GSK concentration used above was 100 nM. Values determined by densitometry show the fold-change in SOX9 and Aggrecan band intensity of each samples. (B) Densitometry of SOX9 and Aggrecan. Values are mean ± SD. αICAM-1: anti-ICAM-1 neutralizing monoclonal antibody, αCD44: anti-CD44 neutralizing monoclonal antibody.

We examined the effect of the inhibition of the association between the endogenous pericellular HA and the receptors for HA located on the cell membrane. Pre-treatment with the neutralizing antibody against ICAM-1 significantly decreased the mRNA expression levels of SOX9 and Aggrecan induced by TRPV4 activation in a dose-dependent manner (Fig 5A and 5B). However, the ICAM-1 neutralization did not suppress the mRNA expression levels to the untreated control levels. Although the inhibition of CD44 demonstrated the similar effect on the SOX9 and Aggrecan mRNA expression, the suppressive effect was not statistically significant (Fig 5C and 5D). The protein expression levels of SOX9 and Aggrecan also determined by the western blotting analysis. Both the anti-ICAM-1 and anti-CD44 neutralizing antibody suppressed the SOX9 and Aggrecan protein expression levels in a dose dependent manner (Fig 6A and 6B). However, the expression levels were not decreased to the untreated control levels. These results suggest that the association between endogenous pericellular HA and each ICAM-1 and CD44 partially contribute to chondrogenesis induced by TRPV4 activation.

## Discussion

In this study, pericellular HA was reported to be involved in TRPV4-induced chondrogenesis via association with both ICAM-1 and CD44. This study represents the first systematic investigation of the interaction between HA and TRPV4 activation in chondrogenesis. Our findings can be summarized as follows: (1) TRPV4 activation increased the expression of SOX9 and Aggrecan, which are key factors in chondrogenesis; (2) pericellular HA is required for TRPV4-induced chondrogenesis; and (3) Both ICAM-1 and CD44 were the primary HA receptors involved in chondrogenesis. These results clearly suggest that pericellular HA associated with ICAM-1 or CD44 is crucial for the functioning of TRPV4 during chondrogenesis; however, further studies will be necessary to determine the exact molecular mechanisms.

Several studies have indicated that HA or TRPV4 stimulates chondrogenesis through multiple mechanisms [21, 24–26]. Muramatsu et al. reported that TRPV4 activation with 4α-PDD increased the expression of SOX9 and Aggrecan in the murine chondrogenic cell line [24], which is consistent with our findings. Until recently, the TRPV4 agonist, 4α-PDD, has been generally used to activate TRPV4 function [24, 27, 28]. In the present study, a more potent pharmacological activator (GSK) was introduced in ATDC5 cells for the first time [29, 30]; indeed, our results confirmed that TRPV4 activation enhanced the expression of chondrogenic markers. Another study reported that GSK had no effect on the mRNA expression levels of SOX9 and Aggrecan [21]. This discrepancy may be due to variations in the differentiation process of target cells; particularly, TRPV4 may have more potent effects on the gene expression of SOX9 and Aggrecan in chondroprogenitors than in fully differentiated articular chondrocytes.

We studied SOX9 and aggrecan expression levels as the representative genes evaluating chondrogenesis progression since the COL2 mRNA expression level did not significantly increase within 72 hours. Previous study demonstrated that COL2 expression level significantly increased after day 7 [31]. In the current study, we would observe the increased COL2 expression level in the extended time point.

The SOX9 expression was quickly increased by the GSK stimulation at 6 hours in the current study. Caron, M. M. et al. reported that SOX9 expression was transiently induced at 1–4 hours and steadily increased again from day 7 [31]. We might observe this bimodal expression pattern if we study longer time.

Previous studies have demonstrated that HA-related intracellular signaling is mediated by CD44 and ICAM-1[8, 9]. These two HA receptors have distinct entry points in the intracellular cascade that constitutes the chondrogenic pathway [16, 32, 33]. Hirabara et al. have demonstrated that the inhibitory effect of HA on LPS-induced cathepsin K expression is mainly CD44-dependent and that LPS-induced MMP-1 expression is dependent on CD44 and ICAM-1 signaling [32]. In the present study, each of ICAM-1 and CD44 were involved in but ICAM-1 seemed to have more potent impact on TRPV4-activated chondrogenic signaling. These findings suggest that the extent to which CD44 or ICAM-1 is involved in chondrogenesis may vary according to the elicitor of this response.

Lisignoli et al. previously reported the anti-apoptotic effect of hyaluronan (HA) in human articular chondrocytes [9]. The anti-apoptotic effect of HA was significantly inhibited by both anti-CD44 (57 ± 12% inhibition) and anti-ICAM-1 (31 ± 22% inhibition) monoclonal antibody. They also reported that the mixture of the 2 antibodies had an additive effect, since the inhibition rate increased to 87 ± 13%. We may have the additive effect in our current study when we use anti-CD44 and anti-ICAM-1 antibody simultaneously.

It remains necessary to determine the molecular mechanisms that mediate interactions between HA, its receptors, and TRPV4 during chondrogenesis. Previous studies have demonstrated that HA and its receptors induce sequential activation of the RhoA protein [34–36]. Additionally, Adapala et al. demonstrated that TRPV4 is a critical modulator of Rho activity [18], which has been reported to play a stimulatory role in chondrogenesis [37]. In these experiments, when RhoA inhibitors were added, the chondrocyte-specific genes were downregulated. Thus, all these findings raise the possibility that HA, its receptors ICAM-1 and CD44, and TRPV4 have an interactive effect on Rho-associated signaling pathways; this hypothesis merits further investigation in future studies. Another major limitation of study is a lack of data using the 3-dimensional (3D) cultured cells. We unfortunately failed to establish the 3D culture system using ATDC-5 cells in this study. However, it must be necessary to determine the reproducibility of the current data using 3D cultured ATDC-5 cells in the future.

In summary, for the first time, interactions between HA, its receptors ICAM-1 and CD44, and the TRPV4 cation channel in chondrogenesis have been preliminary suggested. Although the detailed molecular mechanisms should be examined in the future, these findings may suggest the existence of a molecular mechanism that underlies interactive effects of HA and mechanical stress on joint chondrogenesis.

## Supporting information

S1 Fig Cytotoxicity screening of GSK1016790A (GSK) at different concentration and different incubation time in ATDC5 cells. Incubation with 10000 nM of GSK for 48 and 72 hours significantly decreased the cell viability determined by MTS assay using Celltiter 96 Aqueous One Solution Cell Proliferation Assay (Promega). However, 100 nM for 72 hours condition did not affect the cell viability. Values are mean ± SD.
(TIF)

S2 Fig Western blotting analysis showing SOX9 expression in ATDC5 cells, appeared in Fig 4A. (JPG)

S3 Fig Western blotting analysis showing *Aggrecan* expression in ATDC5 cells, appeared in Fig 4A. (JPG)

S4 Fig Western blotting analysis showing SOX9 expression in ATDC5 cells, appeared in Fig 6A. (JPG)

S5 Fig Western blotting analysis showing *Aggrecan* expression in ATDC5 cells, appeared in Fig 6A. (JPG)

S6 Fig Western blotting analysis showing SOX9 expression in ATDC5 cells, appeared in Fig 6D. (JPG)

S7 Fig Western blotting analysis showing Aggrecan expression in ATDC5 cells, appeared in Fig 6D. (JPG)

S1 File Minimal data set for graphs in each Figures and S1 Fig. (XLSX)
