# Supplementary figures and images for: Hyaluronan promotes TRPV4-induced chondrogenesis in ATDC5 cells
Source: PLoS One. 2019 Aug 8;14(8):e0219492. doi: 10.1371/journal.pone.0219492 (PMC6687147; doi:10.1371/journal.pone.0219492)

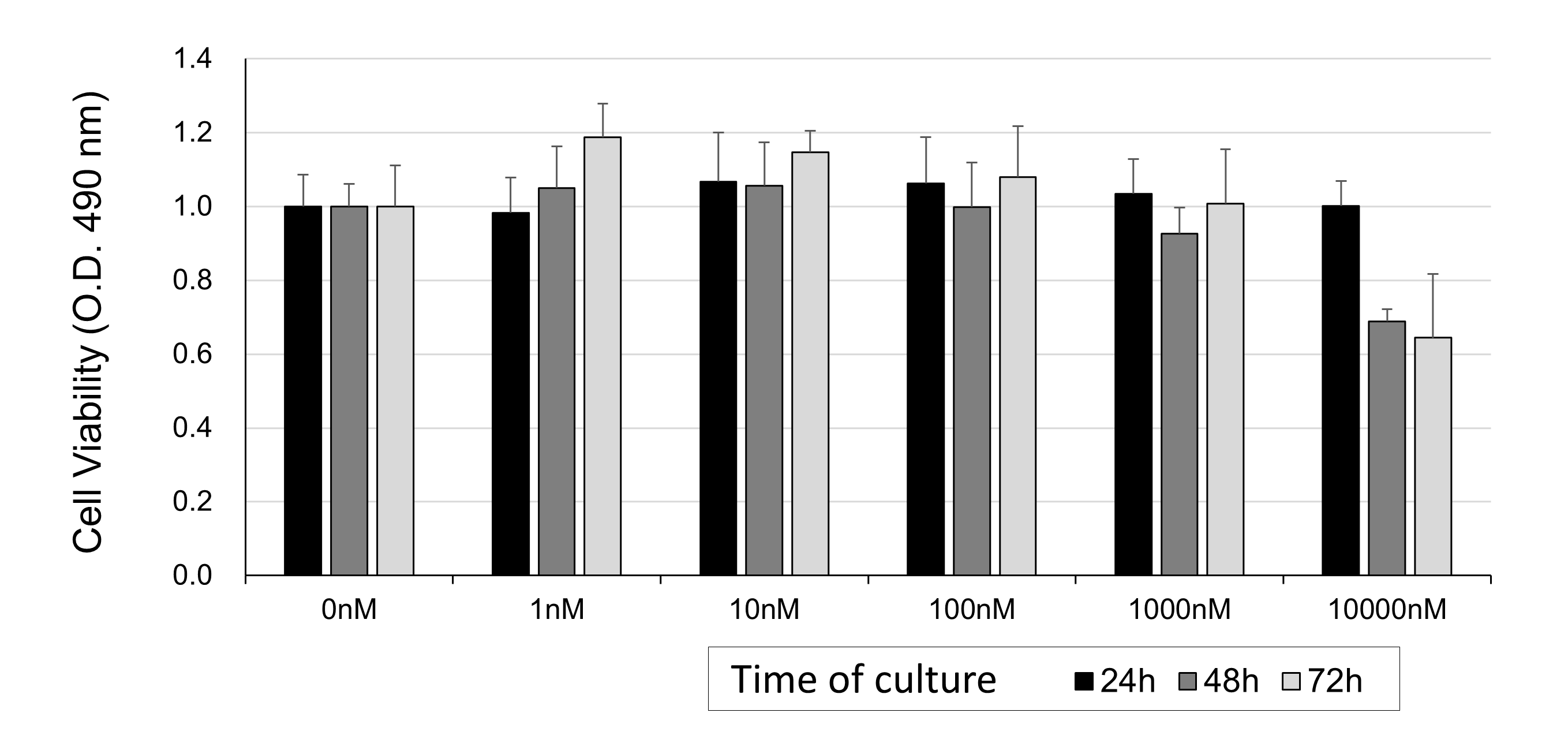

Supplement: S1 Fig — Incubation with 10000 nM of GSK for 48 and 72 hours significantly decreased the cell viability determined by MTS assay using Celltiter 96 Aqueous One Solution Cell Proliferation Assay (Promega). However, 100 nM for 72 hours condition did not affect the cell viability. Values are mean ± SD. (TIF) [file pone.0219492.s001.tif]

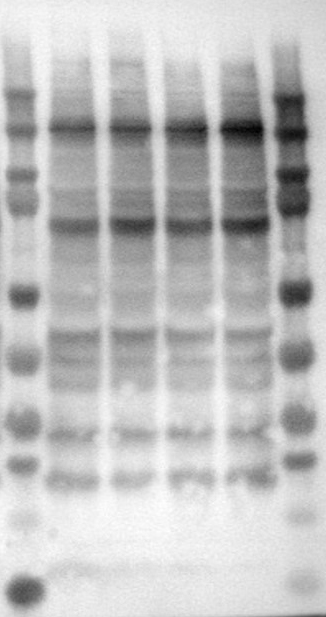

Supplement: S2 Fig — (JPG) [file pone.0219492.s002.jpg]

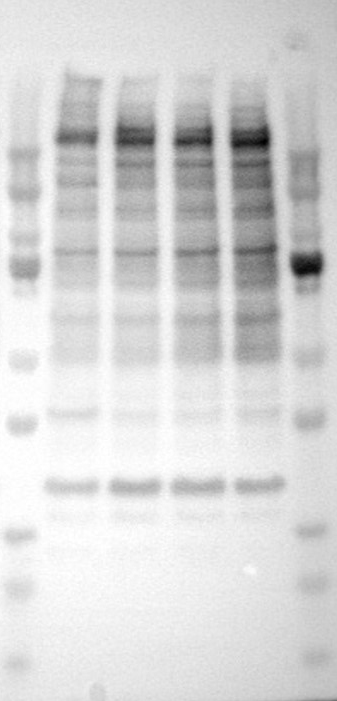

Supplement: S3 Fig — (JPG) [file pone.0219492.s003.jpg]

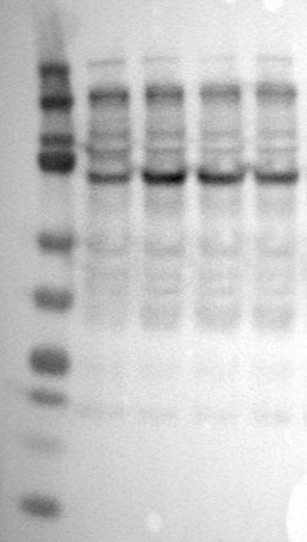

Supplement: S4 Fig — (JPG) [file pone.0219492.s004.jpg]

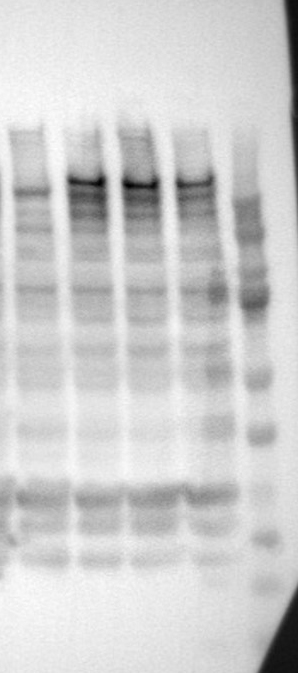

Supplement: S5 Fig — (JPG) [file pone.0219492.s005.jpg]

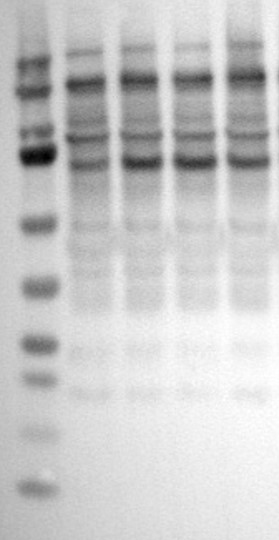

Supplement: S6 Fig — (JPG) [file pone.0219492.s006.jpg]

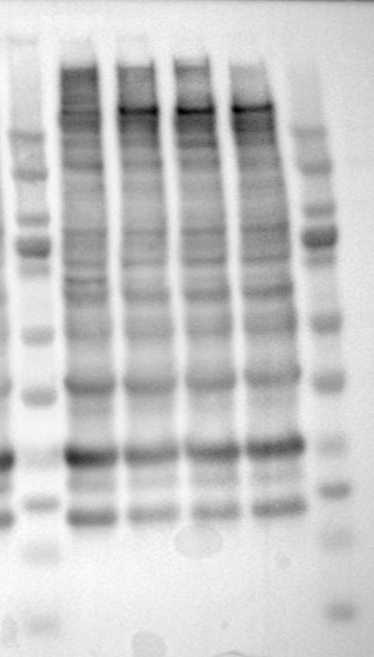

Supplement: S7 Fig — (JPG) [file pone.0219492.s007.jpg]
